# Neglected tropical diseases: recent trends and challenges associated with its rapid diagnosis and effective therapeutic strategies

**DOI:** 10.3389/fphar.2026.1742086

**Published:** 2026-01-22

**Authors:** Subrat Kumar, Abdullah Alghamdi, Puneet Kumar Singh, Nirmalya Pal, Ritesh Pattnaik, Mohammed Alissa, Suad A. Alghamdi, Ghadah S. Abusalim, Subhra Subhadra

**Affiliations:** 1 School of Biotechnology, KIIT Deemed to be University, Bhubaneswar, Odisha, India; 2 Department of Medical Laboratory, College of Applied Medical Sciences, Prince Sattam bin Abdulaziz University, Al-Kharj, Saudi Arabia; 3 ICMR-Regional Medical Research Centre, Bhubaneswar, Odisha, India

**Keywords:** diagnostics, endemic, neglected tropical diseases, one health, pandemic, WHO

## Abstract

Neglected tropical diseases (NTDs) are a distinct group of illness that are primarily prevailing in the tropical regions. NTDs are caused by diverse pathogens including viruses, bacteria, parasites, fungi and toxins, resulting in adverse health, social and economic outcomes. Currently, more than one billion people globally are affected with NTDs, therefore, precise and rapid diagnostic mechanisms are integral for detection and control of NTDs. However, the NTDs programs are underinvested in the progression and enhancement of diagnostic tools. Due to this reason, WHO has released a new road map for NTD 2021–2030 and has pinpointed diagnostics as one of the precedence areas that require concrete action. In order to achieve the 2030 targets, WHO has also established Diagnostic Technical Advisory Group (DTAG) which will help in initiating collaboration among nations to drive advancement in this area. In this review, we explored the epidemiology and burden of NTDs, the challenges in their mitigation, and the available therapeutic interventions for managing these diseases. We have also highlighted the need to holistic approach like “One health” for an effective elimination of NTDs in affected areas. Elimination of NTDs will enhance the socioeconomic levels of the affected regions, thereby assisting in the accomplishment of few sustainable development goals. Thus, there is a need for worldwide commitment for funding to develop fast and safe therapeutic and diagnostic strategies for NTDs.

## 1 Introduction

NTDs comprise a heterogeneous group of bacterial, parasitic, viral, and fungal infections that predominantly occur in developing tropical and subtropical regions, where poverty remains widespread. Collectively, these 21 diseases affect more than one billion individuals worldwide and disproportionately burden vulnerable populations in resource-limited settings (Tidman et al., 2021). Notably, these same communities face the most severe impacts of global change, particularly climate change (Bryson et al., 2020). The transmission of many NTDs involves complex ecological interactions between humans and a range of vertebrate hosts (e.g., dogs, livestock, snakes) and invertebrate vectors (e.g., mosquitoes, flies, snails), with dynamics strongly affected by environmental variability. Climatic agents like temperature, rainfall, and humidity, as well as extreme weather incidences, modulate disease epidemiology. For example, temperature governs vector reproduction, metabolism, survival, pathogen replication, and the spatial allocation of hosts and vectors, while rainfall enhances vector proliferation by creating suitable breeding habitats (Huang et al., 2019). Conversely, extreme meteorological incidences like, floods or heatwaves, can disintegrate ecosystems, disrupting habitats of both vectors and hosts and enhancing their mortality (Meher et al., 2025).

Beyond ecological drivers, human displacement in areas like sub-Saharan Africa, Southeast Asia, and Central and South America further exacerbates disease vulnerability. While displacement often arises from armed conflict and political instability, it is increasingly driven by climate-induced food and water insecurity, including prolonged droughts. Collectively, population movement, limited healthcare infrastructure, and resource scarcity are expected to intensify existing disease burdens, alter epidemiological patterns, and expand the geographical distribution of NTDs, thereby placing additional populations at risk and compromising ongoing control programmes (Mohapatra et al., 2025). NTDs are characterised by their substantial health, social, and economic consequences, including chronic disability, stigma, and entrenched poverty, despite being largely preventable and treatable. The World Health Organization (WHO) now recognises 21 NTDs, with noma recently added to the list in December 2023 (Mohapatra et al., 2024) (Table 1)

**TABLE 1:** List of 21 NTDs with summary of their global burden and major treatment/Control strategies employed for each. (Data collected from WHO and CDC websites).

S. No	Name of the disease	Global disease burden	Major treatment/Control strategies
1	Soil-transmitted helminthiases (ascariasis, trichuriasis, hookworm)	∼1.0–1.5 billion people infected historically; hundreds of millions of children at risk/large contribution to NTD morbidity. Estimates vary by source	Preventive chemotherapy (periodic albendazole/mebendazole MDA), WASH improvements, health education and targeted case management for heavy infections
2	Schistosomiasis (bilharzia)	∼200–250 million people infected (order-of-magnitude). Major DALY contributor among helminthiases	Preventive chemotherapy (praziquantel MDA), snail control/vector management, WASH, diagnosis and individual treatment
3	Lymphatic filariasis (LF)	Hundreds of millions at risk; tens of millions infected historically with millions suffering chronic disease (lymphoedema, hydrocele)	Annual MDA (ivermectin + albendazole or DEC combos depending on setting), vector control, morbidity management and surgery (hydrocele repair), WASH.
4	Onchocerciasis (river blindness)	Tens of millions infected historically; many in sub-saharan africa. Significant morbidity from blindness/skin disease	Community-directed MDA (ivermectin), vector control (blackfly control), surveillance, ocular care
5	Trachoma	Historically tens of millions with active infection; major cause of infectious blindness in endemic areas	SAFE strategy: Surgery (for trichiasis), antibiotics (azithromycin MDA), facial cleanliness, environmental improvement (WASH)
6	Leprosy (hansen disease)	Global new case detection in the tens of thousands–low hundreds of thousands per year (declining), with pockets of higher incidence. Can cause chronic disability	Early diagnosis and multi-drug therapy (MDT: Rifampicin, dapsone, clofazimine), reconstructive surgery, prevention of disability and rehabilitation
7	Visceral and cutaneous leishmaniasis	Visceral leishmaniasis: Tens of thousands of cases per year (major mortality if untreated); cutaneous forms far more numerous in some regions	Case detection and treatment (amphotericin B, miltefosine and other agents depending on species), vector control (sandfly control), reservoir control, improved diagnostics
8	Chagas disease (american trypanosomiasis)	Millions infected in Latin America (chronic infections can cause cardiomyopathy); global burden includes migrants	Antiparasitic therapy (benznidazole, nifurtimox) for acute and some chronic cases, vector control (reducing triatomine insects), blood-supply screening, congenital screening
9	Human african trypanosomiasis (HAT/sleeping sickness)	Cases decreased dramatically; hundreds–few thousands of cases/year during recent years (major progress toward elimination)	Active case detection, treatment (new oral treatments like fexinidazole for gambiense HAT; NECT, pentamidine, etc.), vector control (tsetse fly), surveillance
10	Guinea-worm disease (dracunculiasis)	Near-eradication: Only handful–tens of cases reported annually in recent years (very low)	No drug/vaccine available, containment, safe water, behavioral prevention, vector control, case containment; surveillance
11	Yaws (endemic treponematoses)	Eliminated in some countries; pockets remain — cases are not huge but elimination is targeted	Single-dose azithromycin mass treatment campaigns (oral macrolide), case detection, water/hygiene
12	Buruli ulcer	Focal outbreaks; case numbers relatively low compared to helminthiases but can cause severe morbidity and disability	Early detection and antibiotic therapy (rifampicin + clarithromycin/streptomycin formerly), surgery for severe lesions, wound care, rehabilitation
13	Rabies (dog-mediated)	Tens of thousands of human deaths annually (majority in asia & africa), mostly dog-mediated; vaccine-preventable	Pre- and post-exposure prophylaxis (human vaccines + immunoglobulin when indicated), mass dog vaccination, dog population management, bite prevention
14	Cysticercosis/Taeniasis	Neurocysticercosis is a major cause of epilepsy in endemic areas; millions are exposed in Latin America, africa, asia	Case management (anti-parasitic such as albendazole/pr praziquantel for taeniasis/cysticercosis as indicated), surgery for some lesions, pig vaccination and meat inspection and sanitation
15	Echinococcosis (hydatid disease)	Endemic in pastoral areas; global case counts smaller than helminthiases but can cause severe morbidity and surgery-needing lesions	Surgery, percutaneous treatments, albendazole therapy, dog deworming and livestock management
16	Food-borne trematodiases (e.g., liver flukes)	Affects tens of millions in asia and other regions; significant morbidity (liver, biliary disease)	Antiparasitic treatment (praziquantel), food safety measures (cooking fish/vegetables), sanitation and health education
17	Scabies	Hundreds of millions affected globally (common skin infestation). Severe complications from secondary bacterial infection in some settings	Mass drug administration with ivermectin in high-prevalence communities, topical permetherin for individuals, improved hygiene and housing and management of secondary infections
18	Dengue	Millions of symptomatic cases and tens of thousands of deaths annually in endemic areas; large epidemic potential; major burden in asia & Latin America	Case management (fluid resuscitation, supportive care), vector control (*Aedes aegypti* control), vaccine use in limited settings (dengvaxia and newer vaccines with indications)
19	Chikungunya *(often grouped with Dengue)*	Explosive outbreaks in many regions; many millions infected in recent epidemics (episodic)	Supportive clinical care, vector control (aedes control), surveillance; no widely used licensed antiviral—vaccines under development
20	Mycetoma, chromoblastomycosis and other deep mycoses	Mycetoma and chromoblastomycosis are focal, chronic, highly disabling; case numbers are smaller but major local morbidity. WHO added mycetoma as an NTD in recent years	Prolonged antifungal/antibacterial therapy, surgery for advanced lesions, early detection and rehabilitation, community education
21	Snakebite envenoming	Causes tens of thousands of deaths per year and many more injuries/disabilities, mainly affecting rural tropical communities	Timely antivenom therapy, emergency care and supportive treatment, strengthening supply chains for antivenoms, community education and preventive measures
22	Noma	140,000 cases per year, primarily from sub-saharan africa, high mortality rate (90% if untreated)	Treatment with amoxicillin and metronidazole with nutritious food. Maintain oral hygiene and regular wound care

The inclusion and prioritisation of NTDs have been shaped by WHO’s Global Plan and successive strategic frameworks, which determine the allocation of global attention and funding. Earlier lists encompassed conditions such as anthrax and Japanese encephalitis; however, these were later removed. By 2016, WHO had consolidated a core list of 17 NTDs, which was subsequently expanded to 20 with the addition of foodborne trematodiases, mycetoma and other deep mycoses, scabies and other ectoparasites, and snakebite envenoming. With the most recent addition of noma, the priority list now comprises of 21 NTDs. Importantly, this list remains dynamic, allowing for the inclusion or removal of diseases in response to emerging evidence, shifting disease burdens, and evolving public health priorities (Hietanen et al., 2025).

According to a World Bank analysis, 51% of the population in sub-Saharan Africa, which carries the major share of the NTD burden, survives on less than US$1.25 per day (Mitra and Mawson, 2017). As per the 2010 Global Burden of Disease study, it is estimated that NTDs accounted for 26.06 million disability-adjusted life years (DALYs) (Hotez et al., 2014). Beyond their direct health consequences, NTDs impose severe social and economic costs, including stigma, physical disability, disfigurement, blindness, discrimination, malnutrition, growth impairment, and cognitive deficits. These outcomes reinforce poverty by limiting productivity, constraining opportunities, and undermining the wellbeing of families, communities, and nations.

Despite their impact, many NTDs are avoidable and could be prevented through enhanced sanitation, vector control, effective treatments, and mass drug administration (MDA) programmes. From a realistic perspective, the WHO classifies NTDs into two categories: (i) preventive chemotherapy and transmission control (PCT) NTDs, and (ii) innovative and intensified disease management (IDM) NTDs (WHO, 2024). PCT NTDs such as lymphatic filariasis, onchocerciasis, schistosomiasis, and soil-transmitted helminthiasis are addressed primarily through the periodic distribution of safe, effective, and low-cost (often donated) medicines to populations at risk. IDM NTDs, including Buruli ulcer, Chagas disease, human African trypanosomiasis, and leishmaniasis, require alternative strategies, as large-scale tools for their control remain inadequate (Rosenberg et al., 2016). Change in climatic conditions and global warming are projected to amplify the transmission of multiple vector-borne illnesses, like malaria, dengue, Chagas disease, leishmaniasis, filariasis, onchocerciasis, schistosomiasis, and trypanosomiasis (Tidman et al., 2021). Recognizing the urgent need for innovation in mitigating NTDs, the World Intellectual Property Organisation (WIPO) launched a 5-year roadmap in May 2017 for strengthening research collaborations, capacity building, and outreach programs for NTDs, along with malaria, and tuberculosis, that together affect the world’s poorest populations (World Intellectual Property Organization, 2017).

The most recent NTD roadmap, given by WHO, outlines global targets for preventing, controlling, eliminating, and eradicating NTDs by 2030. It also highlights the necessity of identifying current and emerging trends to guide programme design and its implementation (Souza et al., 2021). Achieving these goals will require coordinated multisectoral action to address climate change alongside other major risks, including epidemics, political instability, migration, and antimicrobial resistance. The intent of this review is to analyze current trends in NTDs focusing specifically on diagnostic methodologies and the associated challenges that influence disease detection and subsequent policy development. The review also aims to highlight various advancements made in the field of detection and diagnosis of NTDs that will help in developing safe, effective and rapid assays and therapeutic agents for NTDs. Anticipating that these developments will be essential for predicting disease distribution and enabling timely, targeted public health interventions.

## 2 Methods

The veracity and replicability of this review article was identified through a systematic search of several major academic databases, specifically focusing on PubMed, Scopus, and Web of Science. The search was limited only to English literature published between 1 January 2015 till 31 October 2025, to ensure the relevance of the findings. Key search terms were developed from core research concepts and included (but were not limited to): “Neglected tropical diseases” OR “NTDs”; “Diagnosis of NTDs” AND” Challenges”; “WHO list of NTDs”; “challenges with NTDs”; “Emerging NTDs”.

Inclusion criteria: Peer reviewed, full-text articles published exclusively in English that directly addressed the research question. Exclusion criteria: We filtered out preprints, conference abstracts, and non-English language publications. After an initial screening of titles and abstracts, full-text review of potentially relevant articles was analysed, data was extracted and synthesized qualitatively to identify comprehensive themes, trends, and key findings in the literature. The entire process adhered to standard reporting guidelines for systematic reviews (PRISMA) to minimize bias and ensure a transparency and replicability.

## 3 Epidemiology

NTDs encompass a distinct group of infectious diseases which are predominantly concentrated in tropical regions of low and middle-income countries, where inadequate sanitation and limited healthcare infrastructure facilitate their persistence (Kurcheid et al., 2022). These diseases pose a considerable threat to global health, social welfare, and economic stability, often leading to chronic morbidity or mortality (Kutikuppala et al., 2023). Their impact extends beyond individual health, perpetuating poverty through reduced productivity, increased healthcare expenditures, and social marginalisation (Chen et al., 2025).

**FIGURE 1:**
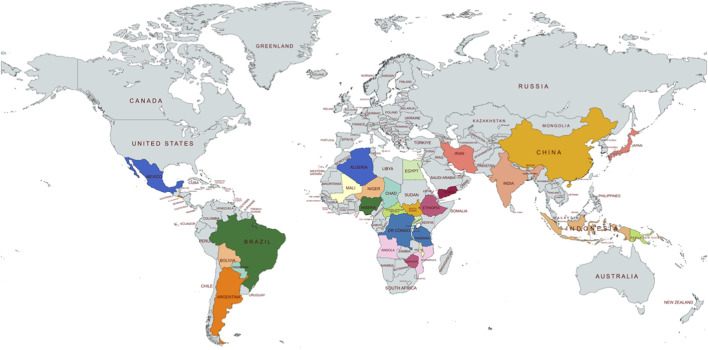
NTD hotspot across the globe.

Globally, NTDs primarily affect populations across Africa, Asia, Latin America, and the Caribbean, accounting for more than 70% of the world’s population (Lin et al., 2022). (Figure 1) The WHO recognised 21 NTDs, including Buruli ulcer, Chagas disease, dengue and chikungunya, dracunculiasis, echinococcosis, foodborne trematodiases, human African trypanosomiasis, leishmaniasis, leprosy, lymphatic filariasis, mycetoma, chromoblastomycosis and other deep mycoses, noma, onchocerciasis, rabies, scabies and other ectoparasitoses, schistosomiasis, soil-transmitted helminthiases, snakebite envenoming, taeniasis/cysticercosis, trachoma, and yaws (WHO, health topics on NTDs).

Transmission routes vary widely among NTDs. Several are vector-borne, which are spread by insects like mosquitoes and flies; prominent examples include malaria, dengue fever, and human African trypanosomiasis (Gangmei et al., 2023). Helminth infections, caused by parasitic worms, are another major category, including soil-transmitted helminthiases (hookworm, roundworm, whipworm) (Akinsanya et al., 2021), schistosomiasis (snail-borne) (Hudu et al., 2024), and lymphatic filariasis (mosquito-borne). Protozoan parasites cause diseases such as Chagas disease, leishmaniasis, and African trypanosomiasis. Bacterial infections, including Buruli ulcer and yaws, and viral infections such as dengue and Zika, are also classified among NTDs (Hudu et al., 2024).

NTDs manifest with a broad spectrum of clinical outcomes, ranging from mild symptoms to severe disability and death (Sapkota et al., 2023). Chronic infections often result in malnutrition, anaemia, impaired cognitive development, and diminished economic productivity (Hudu et al., 2024). By limiting physical and cognitive capacity, reducing school attendance, and restricting work opportunities, NTDs perpetuate the cycle of poverty and exert long-term socioeconomic consequences (Gyapong et al., 2024). Control of NTDs requires integrated approaches, including MDA, vector management, improved sanitation, enhanced health education, and expanded access to healthcare services. Multiple organisations, including the World Health Organization (WHO), the Bill & Melinda Gates Foundation, and a range of non-governmental organisations, coordinate efforts to reduce the global burden of these diseases. The WHO “Roadmap for Implementation” provides a comprehensive framework for the control, elimination, and eradication of several NTDs (Hudu et al., 2024).

Nevertheless, major challenges persist, such as limited financial resources, inadequate awareness, emerging drug resistance, and the need for sustained, cross-sectoral collaboration encompassing health, water, sanitation, and education (Hailu et al., 2024). Despite these barriers, notable progress has been achieved. Guinea worm disease, for instance, has been eradicated in most countries and is nearing eradication in Mali, Chad, South Sudan, Ethiopia, and Angola (Hopkins, 2023). Substantial reductions have also been recorded for onchocerciasis (river blindness) and lymphatic filariasis (Debbarma et al., 2023). The epidemiology of NTDs remains complex, with heterogeneous distributions across regions and populations. Effective elimination will require global cooperation, sustained investment in research and development, and strong community involvement (Mwatondo et al., 2023).

**FIGURE 2:**
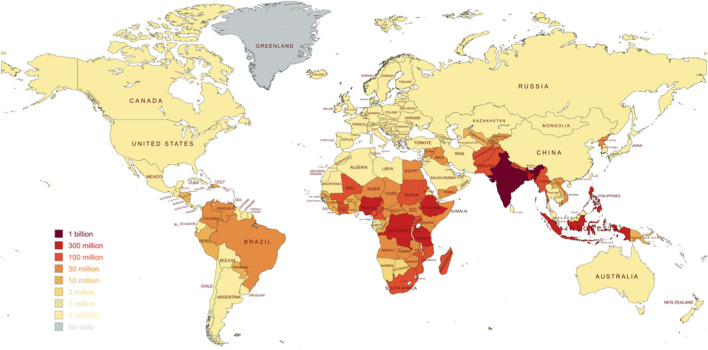
Number of people affected and requiring treatment against NTD in 2023.

Globally, more than one billion people are affected from NTDs, with millions experiencing disability, disfigurement, or premature death as a consequence (Hudu et al., 2024). (Figure 2) These conditions trap affected populations under a cycle of poverty and ill health, undermining both individual wellbeing and broader community development. The economic growth of endemic regions is also severely constrained, as NTDs reduce productivity, increase healthcare expenditures, and disrupt education by lowering school attendance because of illness and disability (Magalhães et al., 2023). Collectively, these impacts reinforce poverty and slow socioeconomic progress.

Many NTDs are co-endemic, meaning that individuals frequently suffer from multiple infections simultaneously. Such overlapping disease burdens exacerbate health outcomes and pose challenges for integrated control efforts. Despite their widespread impact, NTDs have historically received limited consideration from the global health community, the pharmaceutical industry, and research funding bodies. This neglect has restricted investment in new tools, delaying the expansion of more effective treatment and intervention strategies (Hudu et al., 2024).

The burden of NTDs is shaped not only by socio-economic and environmental determinants but has also been profoundly affected by the COVID-19 pandemic, which disrupted both direct and indirect aspects of disease management (Zaidi et al., 2024; Mohapatra et al., 2023). While the pandemic strained health systems globally, the impact was particularly severe in countries with high NTD prevalence, where healthcare infrastructure was already overextended. In India, for example, resource diversion, reduced availability of essential health services for NTD patients, and interruptions in surveillance and data collection significantly undermined control efforts. These challenges underscore the importance of systematically assessing the factors that exacerbated NTD prevalence and incidence during the pandemic, and of translating these lessons into preventive measures to mitigate the effects of future public health crises. Despite the scale of disruption, there remains a notable research gap regarding the impact of COVID-19 on NTD related healthcare services (Zaidi et al., 2024).

The burden of NTDs, commonly expressed in DALYs, integrates years of life lost (YLL) due to premature mortality with years lived with disability (YLD), providing a comprehensive measure of their combined impact on morbidity and mortality (Ferrari et al., 2024). While significant progress has been achieved a 26% decline in the number of people requiring NTD interventions since 2010 an estimated 1.62 billion individuals still require such interventions in 2022. This reduction, although noteworthy, falls short of the WHO road map target of a 90% decrease by 2030, reflecting the influence of complex and fluctuating health, political, and financial constraints.

By 2023, important milestones had been reached: WHO verified 50 countries as having eliminated at least one NTD, representing half the 2030 target of 100 countries. Nevertheless, accelerating progress will require broader engagement from additional countries and greater attention to a wider spectrum of NTDs. To support this, the WHO introduced cross-cutting indicators and the Global NTD Annual Reporting Form (GNARF) in 2023, initiatives that highlighted persistent data quality limitations and systemic challenges in NTD information management (Ferrari et al., 2024).

Temperature and rainfall were the most frequently reported climatic factors influencing vector ecology and disease transmission, while humidity and rising sea levels were also highlighted as contributors to changes in vector distribution (Tidman et al., 2021). Rising temperatures accelerate vector development and increase biting frequency; however, once conditions exceed the thermal tolerance of the vector, growth and survival decline. Elevated temperatures can also shorten viral amplification time, enhancing transmission potential (Fischer et al., 2014).

Increased rainfall can generate new breeding sites for vectors, yet heavy rainfall and flooding may destroy existing habitats (Tidman et al., 2021). Conversely, reduced rainfall may limit vector proliferation, but drought-driven water storage can create alternative breeding sites, thereby facilitating viral transmission (Cunze et al., 2016). Climate-driven sea-level rise may further expand saline and brackish water habitats, favouring the spread of salinity-tolerant vectors (Ramasamy, 2015). Beyond environmental drivers, global travel and trade serve as critical pathways for the introduction of vectors and pathogens into new geographic regions, supporting their establishment and spread. Population growth, migration, land-use change, and rapid urbanisation also amplify transmission risk, particularly in endemic areas. Insufficient sanitation and weak mosquito-control measures are distinguished as additional enablers of disease spread. In contrast, improved housing conditions, better socio-economic development, and effective vector control programmes have been associated with reduced dengue incidence in developed countries (Tidman et al., 2021).

Globally, the COVID-19 pandemic had directly affected the prevention, control, and management of NTDs. As COVID-19 garnered more focus, various NTD programmes suffered disturbances because of reallocation of resources that was originally allocated for NTD eradication programs (Ehrenberg et al., 2021; Mohapatra et al., 2023). Lockdowns, travel restrictions, and the prioritisation of COVID-19 services created substantial challenges for the implementation of MDA programmes (Borlase et al., 2022). Limitations on movement and reduced healthcare facility capacity restricted access to essential services for NTD patients, leading to obstacles in seeking timely diagnosis, treatment, and follow-up care (Hotez et al., 2021). Surveillance and data collection were also disrupted, as field activities like survey`s and assessments were curtailed because of safety issues and logistical constraints (Ehrenberg et al., 2021).

The suspension of non-essential medical procedures further delayed diagnostic testing for NTDs, while interruptions in MDA campaigns, which were crucial to control lymphatic filariasis and soil-transmitted helminthiasis, arose from restrictions on community gatherings and mobilisation (Hollingsworth et al., 2021). Vector-control initiatives, particularly those targeting mosquitoes, were similarly affected by lockdowns and the diversion of resources to COVID-19 response efforts. These disruptions collectively delayed diagnosis, treatment, and preventive measures, raising the risk of complicating the existing NTD cases and slowing progress towards its elimination (de Souza et al., 2020). In India, where NTD prevalence is high, the indirect consequences of the pandemic including financial hardship, reduced healthcare access, and interruption of control programmes likely compounded the overall disease burden (Zaidi et al., 2024).

## 4 Advances in diagnostic technologies

Effective diagnostic techniques are a key point to prevent and control NTDs. WHO released a new road-map for ending NTDs (2021–2030) on ninth of April 2020 (WHO report, 2025). This road-map emphasizes the need for effective diagnostics for NTDs as a pre-requisite for reaching the set disease targets by 2030. For confirmation of disease, screening of population, surveillance, disease monitoring and evaluation diagnostics are a crucial component. Control and elimination programmes for NTDs will be successful with the availability of sensitive and specific diagnostic tools. Traditional diagnostic approaches are time consuming and cumbersome (Table-2).

**TABLE 2:** Diagnostic assays for various NTDs with their limitations and potential improvements.

Disease	Common diagnostic assays	Advantages	Limitations	Potential improvements
Soil-transmitted helminths	Kato-katz thick smear (stool microscopy)	Simple, cheap, quantitative (eggs per gram)	Low sensitivity in mild infections, requires microscopy	Develop more sensitive point-of-care antigen tests; multiplex stool PCR for pragmatic use
Schistosomiasis	Kato-katz (stool/urine), urine filtration, circulating cathodic antigen (CCA) rapid test	Simple and cheap; CCA can be deployed in field	Less sensitive in mild disease; CCA less reliable for some species (e.g., S. haematobium)	Development of species-specific antigen RDTs; integrate multiplex antigen/PCR assays
Lymphatic filariasis	Immunochromatographic card test (ICT) for circulating filarial antigen, Og4C3 ELISA, night blood smear for microfilariae	ICT is rapid and field-friendly; ELISA is sensitive	ICT less accurate after MDA; blood smears insensitive, require night collection	Next-gen RDTs with higher specificity post-MDA; molecular monitoring of vectors
Onchocerciasis	Skin snip microscopy, Ov16 antibody rapid test	Skin snip can detect active infection; Ov16 RDT is non-invasive	Skin snip invasive and less sensitive at low prevalence; Ov16 cannot distinguish past vs. current infections	Improve antigen detection assays; combine serology with molecular tests
Trachoma	Clinical grading (SAFE strategy), PCR for *Chlamydia trachomatis*	Clinical exam cheap and field-usable; PCR highly sensitive/specific	Clinical exam is subjective nature; PCR is expensive and requires lab and expertise	Develop cheap point-of-care nucleic acid or antigen detection assays
Leprosy	Clinical diagnosis, slit-skin smear microscopy, PCR	Clinical diagnosis feasible in endemic areas; PCR sensitive	Smear microscopy has low sensitivity; serological tests are not reliable	Biomarker-based RDTs for early/subclinical cases
Leishmaniasis (visceral)	rK39 antibody rapid test, direct agglutination test (DAT), PCR	rK39 simple and widely used; DAT sensitive	rK39 less reliable in east africa; antibody tests cannot distinguish active vs. past infections	Antigen detection tests; multiplex PCR for species identification
Chagas disease	ELISA (antibody), rapid antibody tests, PCR, xenodiagnosis	ELISA sensitive; RDTs usable in field	Antibody tests cannot detect cure easily; PCR not standardized for routine use	Standardized antigen/PCR assays; field-deployable tests for congenital screening
Human african trypanosomiasis (HAT)	CATT (serology), microscopy of blood/CSF, molecular assays, rapid serological tests	CATT useful for screening; microscopy confirms active infection	CATT less reliable for rhodesiense HAT; microscopy insensitive for low parasitemia	Better antigen detection; portable molecular assays (e.g., LAMP)
Guinea worm disease	Clinical diagnosis (emergent worm), microscopy for larvae	Simple; easy case recognition	No early-stage diagnostic tool	Antigen detection assays to identify prepatent infections
Yaws	RPR/VDRL (non-treponemal), TPHA/TPPA (treponemal), dual RDTs	RDTs enable rapid field screening	Cannot distinguish yaws from syphilis	Yaws-specific antigen assays
Buruli ulcer	Clinical exam, PCR for IS2404, histopathology	PCR highly specific; clinical diagnosis in endemic areas	PCR requires lab; early lesions mimic other skin conditions	Portable molecular tests; antigen-based RDTs
Rabies	DFA test (gold standard, postmortem brain tissue), RT-PCR, direct rapid immunohistochemical test (dRIT)	DFA/dRIT sensitive and specific; PCR usable in labs	DFA requires brain tissue (postmortem); limited access in endemic areas	Saliva/skin antigen detection; field-deployable lateral flow RDTs
Cysticercosis	Brain imaging (CT/MRI), EITB antibody test, antigen ELISA	Imaging sensitive for neurocysticercosis; EITB highly specific	Imaging costly; antibody tests cannot distinguish viable vs. dead cysts	Antigen detection for active infection; cheaper neuroimaging approaches
Echinococcosis	Ultrasound, CT/MRI, serology (ELISA, immunoblot)	Imaging detects cysts; serology supports diagnosis	Serology variable sensitivity/specificity; imaging costly	Point-of-care imaging (portable ultrasound), improved species-specific serology
Food-borne trematodes	Stool microscopy, serology, PCR	Microscopy cheap; PCR accurate	Microscopy insensitive in light infection; serology cross-reactive	More sensitive stool antigen tests; rapid species-specific molecular tests
Scabies	Clinical exam, dermatoscopy, skin scraping microscopy	Simple in endemic areas; dermatoscopy non-invasive	Microscopy insensitive; exam subjective	RDTs for mite antigen or DNA; AI-assisted image diagnosis
Dengue/Arboviruses	NS1 antigen RDT, IgM/IgG ELISA, RT-PCR	NS1 detects acute cases; PCR is gold standard	Serological cross-reactivity with other flaviviruses; PCR costly	Multiplex RDTs for dengue/chikungunya/Zika
Mycetoma	Clinical exam, culture, histopathology, PCR	Histopathology/Culture confirm diagnosis	Culture slow; PCR not widely available	RDTs for rapid pathogen differentiation
Snakebite envenoming	Clinical syndrome, immunoassays (experimental)	Clinical diagnosis immediate	Lack of species-specific diagnostics delays treatment	Field RDTs for venom antigen to guide antivenom choice

The diagnosis of NTDs, particularly endoparasitic infections, used microscopy for examination of blood, urine and stool samples. It not only relies on in-depth training involving sample preparation but also expertise in reading and interpreting the results of microscopic examination (Cringoli et al., 2010). Also, the sensitivity of microscopic diagnosis relies on the intensity of infection, i.e., the number of parasites or their elements present in the sample making it a major problem in diagnosing sub-clinical or early infections. Immuno-diagnostic assays used for NTDs often lack sensitivity and/or specificity making them prone to error leading to mis-diagnosis. For example, in NTDs caused by helminth cross-reactivity of antibodies is observed (Petralia et al., 2023). A major drawback of global control efforts for control of NTDs is non-availability or inadequate availability of reliable and advanced diagnostic tests. Absence of rapid, easy-to-use yet accurate point-of-care (POC) tests contributes to general neglect of NTDs and the under-appreciation of the burden of disease. Another setback is sample collection for these diagnostic tests which are often patient-invasive. Laboratory exclusive POC diagnostic tests will aid rapid screening of NTDs. POCs developed aiming at minimal processing steps to obtain an easy-to-interpret and robust result with short turn-around time (TAT) is the need of the hour. These should not be labour-intensive and use expensive laboratory procedures/equipments. Advanced POCs have been designed with the integration of advances in the fields of biotechnology, nanotechnology, electrochemical or optical methods and micro-fluidics for example, loop-mediated isothermal amplification (LAMP) assay, lateral flow devices, use of biosensors, etc. (Bharadwaj et al., 2021). Nucleic acid based POCs and qPCR have also proved to be sensitive tools in the diagnosis of NTDs. Recombinase Polymerase Amplification (RPA) technology offers fast, isothermal processing conditions and minimal preparative testing which can be adopted for the diagnosis of NTDs in low-middle-income-countries (LMICs) with limited resources (Hueso et al., 2025). Multiplex real-time PCR assays have been utilised for the differential diagnosis of various NTDs caused by helminths and intestinal protozoan infections (Nurulhasanah et al., 2020; Jasuja et al., 2024)

**FIGURE 3:**
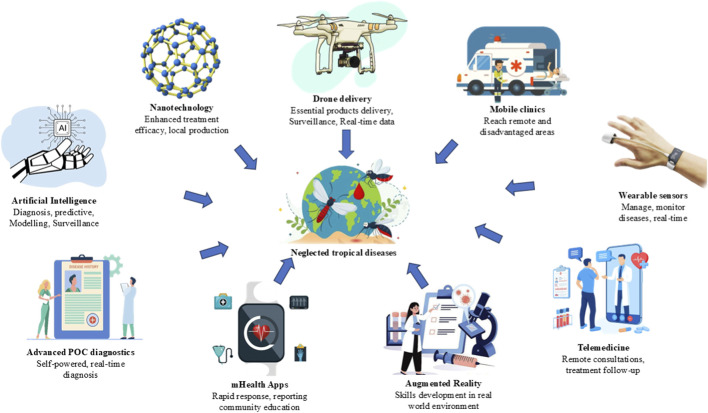
Possible improvements in NTD diagnostics.

Isothermal NAATs, LAMP assays and RPA based techniques allows amplification and detection within a short TAT, i.e., as low as 20 min but assay like these often have low specificity. Novel Clustered Regularly Interspaced Short Palindromic Repeats (CRISPR)-based assays are now being developed for the detection of diseases with increased specificity and sensitivity (Bharadwaj et al., 2021). These assays allow qualitative detection as well as quantification and are highly sensitive. Chamnanchanunt et al. (2020) have reported use of microRNAs (miRNAs) as biomarkers for detection of NTDs (Chamnanchanut et al., 2020) Another advancement in diagnostics is development of easy-to-use devices called as biosensors. These tiny and easy-to-use devices aid in rapid diagnosis leading to a better treatment and faster elimination of NTDs (Cordeiro et al., 2021). The increasing demand for the eradication of deadly NTDs will not rely on any one diagnostic technique but it will need a well-planned and coordinated effort globally involving the use of an array of diagnostic techniques effective for screening, confirmation and elimination of the disease. Diagnostic assays alone or in combination offering high throughput and easily adaptable to remote field conditions will enable accurate diagnosis and epidemiological surveillance of various NTDs. Use of artificial intelligence (AI) has allowed achievement of success in vision tasks and planning. While screening large populations for NTDs AI/deep learning tools can be adopted to analyse vast volumes of data and complex algorithms can be developed to perform specialised tasks. AI can assist healthcare providers in making clinical decisions based on various diagnostic assays. AI should not be treated as a replacement for human diagnosis for various NTDs, but if used appropriately, it can serve as a potent tool that can be used in screening for diseases and improving patient outcomes. AI-powered diagnostic digital tools can become the future of diagnosis of NTDs (Yotsu et al., 2023). (Figure 3)

## 5 Challenges in diagnosis of NTDs

A major challenge in diagnosis of NTDs is the unavailability of an easy to use, rapid andreliable method of diagnosis that can be adopted globally for uniform outcomes. The available diagnostic assays are poorly standardized yielding unspecific and unreliable test results. Available diagnostic assays are not well suited to resource-constrained regions, further adding up to the neglect of such diseases. The COVID-19 pandemic intensely affected global research in healthcare in general and in particular, caused delays and constraints to NTD management and elimination programs. The major challenge in providing molecular testing and automation to resource-limited settings is primarily the cost. Expensive diagnostic assays cannot be adopted globally. Use of multiplex diagnostic assays for the detection of multiple organisms simultaneously can aid in reducing the cost as well as staff requirements. Automation and use of cartridge based-diagnostic tests allows to minimize cost and reduce human errors that often lead to misdiagnosis of NTDs. Large, complex, and high-throughput automated equipment has the drawback in terms of infrastructural requirements, instrument errors and breakdowns that require complex troubleshooting to be performed by specialist technicians (Opota et al., 2020). LMICs often lack the required physical infrastructure, access to refrigerated transport or storage, sterile workspaces, or permanent laboratories to perform specialised diagnostic assays for NTDs (Peeling and McNerney, 2014). Specialised training of staff and technicians is needed for performing diagnostic techniques in the laboratory and this is challenging especially in remote areas. Several NTDs may present sub-clinically or without any profound clinical symptoms which makes it difficult to diagnose clinically. In such cases, the availability of practical diagnostic assays of sufficient sensitivity is needed to detect the levels of infection. The clinical differentiation of arboviral diseases like dengue and chikungunya is a particular challenge. Both diseases present with similar clinical manifestations, especially in early stages of the infection. With the increasing incidence of dengue and chikungunya infections and frequent epidemics being reported worldwide, the availability of reliable diagnostic assays is critical (Karl et al., 2015). Poddighe et al. have emphasised on the significance of accessibility to sensitive diagnostic assays for NTDs to improve disease management in non-endemic settings (Poddighe et al., 2017). This is very essential for early detection and controlling the transmission of the disease. Certain diagnostic assays take more effort in standardizing to laboratory settings in different regions. A more reproducible and well standardized diagnostic assay will help overcome such issues leading to limited diagnosis. Standardized inter-laboratory assay validation, collaboration at the inter-sectorial levels and establishment of a global One Health diagnostic platform, sharing best practices on diagnosis of NTDs, could all substantially contribute to mitigation of these illnesses (Johansen et al., 2015). In order to pave the way for total elimination of NTDs, higher sensitive and specific assays are needed that are easy to use, reproducible and available for deployment in endemic and resource-constrained settings.

## 6 Current and emerging therapeutic strategies

Preventive chemotherapy which is deployed via MDA still remains a primary strategy for a number of NTDs, including lymphatic filariasis, trachoma, soil-transmitted helminthiases, onchocerciasis and schistosomiasis, with the goal of treating at-risk communities regardless of individual diagnosis (WHO, 2024 report). Standard MDA agents for soil-transmitted helminthes (STH) includes mebendazole (500 mg) or albendazole (400 mg); however, ivermectin in combination with albendazole is very efficient against Strongyloides stercoralis and Trichuris trichiura (Chong et al., 2021; Gandasegui et al., 2022). A systematic review and meta-analysis conducted in 2024 demonstrated that ivermectin-based MDA led to an approximate 84% and 50% reduction in the prevalence of Strongyloides stercoralis (threadworm) and T. trichiura respectively and upto approximately 89% reduction when paired with albendazole (Le et al., 2024). Multiple randomised controlled trials indicate that combinations like albendazole plus diethylcarbamazine or ivermectin can considerably lower the frequency of numerous parasitic illnesses, including hookworm, whipworm, elephantiasis, and roundworm (Reddy et al., 2007).

Certain conditions like Human African trypanosomiasis, leishmaniasis, Chagas disease, and Buruli ulcer cannot be treated with MDA. WHO recommends that trained healthcare professionals should treat these conditions under clinical settings using disease-specific medications like liposomal amphotericin B (L-AmB) for treating visceral Leishmaniasis (VL), respectively (Sundar et al., 2019), oral fexinidazole for treating human African Trypanosomiasis (HAT) (Lindner et al., 2025) and an 8-week treatment of Buruli ulcer using streptomycin with rifampicin (Matovu et al., 2025).

Over the years, major concerns have been raised over the safety of these drugs for treating NTDs. This has been further mired with the rise of drug-resistant pathogens, such as asthelmintic resistant soil transmitted helminths, threatening the efficacy of MDA programs and needs timely alternative regimens and resistance surveillance (Ng’etich et al., 2024). Some protozoan NTDs, like those caused by Leishmania sp., can change and adapt the genetic makeup which makes them resistant to drugs and difficult to treat (Bhusal et al., 2025). Similarly, African trypanosomes which has become resistant to suramin and arsenicals, should be treated fexinidazole (Mordt et al., 2022). Although, Benznidazole still remains the principal treatment for Chagas disease, it is not always effective in long-term infections. Studies have shown that in some cases, the parasite may become less responsive to the drug. Thus, combination therapy should be explored for better prognosis (Porta et al., 2023). Standard antibiotics treating bacterial NTDs, such as rifampicin for Buruli ulcer, are not always successful, thus requiring alternative treatment strategies (Muhi et al., 2025). For fungal NTDs like eumycetoma, treatment often involves taking azole medicines for months, which may not provide a complete cure and can have side effects. Further, it has been noticed that some fungal strains can become drug resistant, making their treatment harder. Studies on an oral drug called fosravuconazole suggests that it might be easier to tolerate and could work better, but it has its own concerns (Fahal et al., 2025). Currently, majority of NTDs with viral aetiology (e.g., rabies and dengue) are treated with preventative and supportive care strategies rather than any particular antiviral medication (Obi et al., 2021). There is no commonly used, standardised antiviral therapy for most viral NTDs, and vector control plays an important role in prevention.

Since last few decades, major advancements have been made in nanotechnology, drug delivery strategies and development of novel therapies like mRNA. Computer-based approaches and high-throughput screening have helped to speed up the search for new treatments for NTDs (Hernandez et al., 2018). In some cases, drugs that are already been licensed for other diseases are being used and tested against these infections, making it faster and easier since their effects and safety have already been studied (Hernandez et al., 2018). Nanoparticle-based drug formulations have been evaluated for the treatment of Leishmaniasis and Chagas, while lipid-based carriers have been used to deliver ursolic acid for visceral leishmaniasis. These strategies have improved bioavailability, drug solubility, and efficacy (Mengarda et al., 2023). Combination therapies are being tried for treating fungal NTDs such as eumycetoma to reduce the side effects, while boosting the immune system (Farid et al., 2025). Repurposing of drugs has added more treatment options, still drug resistance remains the major hurdle for treating NTDs (Ferreira et al., 2022).

## 7 Challenges in therapeutic implementations

In many endemic regions where NTDs are common, lack of reliable health data, weak monitoring, and poor primary healthcare support make it hard to scale-up of NTD services. Recent studies suggest that including NTD care as a routine part of health services is possible, but progress depends on proper supervision, strong involvement from local communities and on sustained training (Donovan et al., 2025; Hailu et al., 2024). Getting supplies and medications where they are needed is still a big problem. Integrating NTD drugs into a country’s regular health system can help with delivery and storage, but there are still issues with reaching remote areas and predicting the demand especially when programs are interrupted (Kollie et al., 2023; Itaye et al., 2023).

Affordability makes access difficult. A recent study showed that while the cost of albendazole dropped by about 78%, the cost of albendazole went up by around 60%. Some drugs, like miltefosine, remain very expensive, and since only a handful of companies produce them, the chances of shortages are high (Goh et al., 2025).

MDA does not always reach people consistently across different places and years even when medicines are available. This shows the need for stronger support from the health system and the need for delivery strategies that fit local conditions (Namara et al., 2025). Cultural factors play a critical role in whether people accept treatment. Studies showed that when there is trust in those giving clear information, the medicines, and the program fits well with local life, more people take part. But language barriers, worries about side effects, weak involvement of the community leaders and constant movement of groups like herders or migrants often lead to low participation and leave some groups untreated (Amanyi-Enegela et al., 2024; Kimani et al., 2025; Mitchell et al., 2022).

Regulations and policies can either slow down or support the progress. Experts suggest that community involvement and inclusive national policies are needed to keep treatment programs going. At the same time, regulatory routes like the U.S. FDA’s special programs for tropical diseases can speed up access to vaccines, drugs, and tests, provided countries can also fund and adopt them (George et al., 2023; Mukherjee, 2023). Thus, to make progress, efforts must focus on affordable access to medicines, strengthening routine healthcare services, creating policies and rules that support fairness as the program grows, and delivering care in ways that fit local cultures.

## 8 Integrated approaches and global initiatives

On the second World neglected Tropical Diseases Day, which was celebrated on 30th January 2021, WHO launched its NTDs roadmap for 2021 to 2030 (World Health Organization, 2021a). It has set up certain targets such as 90% reduction in the number of people requiring treatment against NTDs, 75% lowering of DALYs related to NTDs, 100 countries having eradicated at least 1 NTD and at least 2 NTDs eliminated globally. These targets will help in achieving the sustainable development goals. WHO has developed this roadmap after extensive global consultations with all NTDs stakeholders and the document has been endorsed by 194 member states (World Health Organization, 2021b). In order to achieve these targets, the roadmap also describes an integrated approach to develop new interventions and affordable diagnostics and integration of these interventions in national health coverage for a targeted 75% reductions in mortality due to NTDs. WHO also aims to establish an NTD diagnostic technical and advisory group (DTAG) in order to provide guidance on strategies for eradication programmes and to manufacture targeted product profiles for precedence diagnostics (World Health Organization, 2020a).

Due to the ongoing COVID-19 pandemic in tropical and sub-tropical regions, a lot of these plans meant for NTDs were jeopardized. These disruptions might be responsible for an elevated infection and death associated with NTDs and delays in attaining the goals set by the 2021–30 roadmap. Further the COVID-19 pandemic has raised challenging questions like the accessibility of vaccines to people from low-income countries (World Health Organization, 2020b). In order to overcome this vaccine treaty like “GAVI”, Coalition for Epidemic Preparedness Innovations (CEPI) were initiated that partnered with WHO, UNICEF, World Bank and Gates foundations to strengthen the PHCs and taking us closer to sustainable development goal of universal health coverage (Dyer, 2020; World Health Organization, 2021c). Apart from these global initiatives, public-private partnerships and NGOs can play a crucial role in our fight against NTDs, as they can facilitate collaboration between governments, corporate organisation and other stakeholders to develop innovative and effective medical solutions for NTDs (Ma et al., 2023). NGOs can work directly with the communities and can build trust and raise awareness, ensuring that NTDs mitigation strategies are tailored to local needs. They can also provide direct services like, MDA, vector control, and health education, especially in areas where healthcare is limited. Various such NGOs are the Damien foundation, sightsavers, PCI India and the task force for global health who are engaged in controlling NTDs (Aerts et al., 2017). Thus, in order for an effective and sustainable control of NTDs, we require a strong collaboration between PPPs, NGOs and as well as with governments, international agencies and other stakeholders. This collaboration must ensure that resources are efficiently used, interventions are reaching to those who need them most and our progress towards controlling and elimination NTDs is accelerated.

## 9 Future perspective and recommendations

Enhancing diagnostic innovation and its reach for neglected tropical diseases (NTDs) is very important, since many cases go unnoticed because reliable tests are difficult to get in the endemic regions where these illnesses are common. Advances in portable sequencing platforms and point-of-care molecular diagnostics could help in detecting diseases with better accuracy and sooner, even in remote areas (WHO, 2023).

To complement this, accelerating therapeutic pipeline development will need more than just developing new therapeutics. It also calls for repurposing the existing drugs, new tools like advanced biologic therapies and nanotech. Stronger teamwork between global private and public sectors is crucial to tackle the longstanding neglect in NTD drug research and development (Ahmed et al., 2022).

At the same time, strengthening surveillance and data sharing is crucial to track rising drug resistance, changes in disease carrying vectors, and new outbreaks, many of which are being affected by climate change. Using regional data sharing platforms and genomic epidemiology is becoming an important way to strengthen NTD monitoring (WHO, 2025). With the arrival of Artificial Intelligence, its integration within the One Health framework offers a synergistic and comprehensive strategy for the surveillance, diagnosis and control of NTDs, which are often zoonotic and heavily influenced by environmental factors (Manyazewal et al., 2024; Vaisman et al., 2020)

Lastly, policy reforms and sustainable financing models are important for lasting progress. Better cooperation between countries, making NTD care a part of universal health services, and new ways of financing are all important to keep efforts sustainable and fair in the long run (Rahman et al., 2025). Thus, together these steps build reliable and long-lasting public health solution against the fight for NTDs.

### 9.1 Limitations

Although we have tried to carry out an overview of NTDs with emphasis on their diagnostic challenges, it has many potential limitations. First, the article sourcing process was restricted to online databases like Scopus, Pubmed and WOS, but no other databased were searched. Therefore, additional relevant studies might have been missed. Second, we included only studies published in English within a specified time frame, which may have led to the exclusion of relevant literature published in other languages or earlier periods. This language and temporal constraint potentially narrow the scope of evidence and may introduce publication bias. Third, the overall validation scope of this review remains low, given the heterogeneous methodologies and varying quality of the included studies. Fourth, the predominantly regional concentration of research—originating largely from specific geographical areas—restricts the generalizability of the findings to a broader global population. Finally, we have excluded articles published in preprint databased due to the lack of peer review. These limitations highlight the need for more diverse, comprehensive, and methodologically consistent future research to validate and expand upon the insights presented in this review.

## 10 Conclusion

Neglected tropical diseases (NTDs) impose a significant health, social, and economic burden on more than one billion people, primarily residing in low- and middle-income countries across tropical and subtropical regions. Their persistence is shaped by poverty, inadequate sanitation, limited access to healthcare, and environmental conditions that sustain complex transmission patterns. These challenges make it clear that medical interventions alone cannot eliminate NTDs; instead, sustained progress requires a multifaceted approach that includes community engagement, education, capacity building, and improvements in infrastructure. Although therapeutic interventions have advanced disease control, drug resistance, supply chain weaknesses, and inconsistent distribution still limit long-term effectiveness. A comprehensive framework such as the One Health approach offers a promising pathway by recognizing the interconnections between human, animal, and environmental health. This model strengthens disease surveillance and control while enhancing resilience against future outbreaks through cross-sectoral collaboration.

Despite the vast burden posed by NTDs, its diagnostics have received insufficient attention and investment, creating a major barrier to timely detection, management, and elimination. The WHO NTD Road Map 2021–2030 emphasizes the urgent need to expand diagnostic capacity, and the establishment of the Diagnostic Technical Advisory Group (DTAG) marks an important step toward fostering innovation, coordinating international efforts, and integrating new tools into existing health systems. Achieving the 2030 targets will require unwavering global commitment, including strong political will and sustained financial investment. Expanding funding for research and development of rapid, accurate, and affordable diagnostic tools—alongside safe and effective therapeutics—is critical. Aligning NTD elimination efforts with the broader Sustainable Development Goals (SDGs) will further ensure that progress contributes to reducing poverty, improving education, promoting gender equality, and strengthening overall societal wellbeing. In conclusion, combating NTDs is not only a health priority but a developmental imperative. By embracing innovation, enhancing collaboration, and adopting holistic strategies, the global community can advance toward the eradication of NTDs and improve the quality of life for millions worldwide.
